# Remineralization Potential of Nanohydroxyapatite Toothpaste Compared with Tricalcium Phosphate and Fluoride Toothpaste on Artificial Carious Lesions

**DOI:** 10.1155/2021/5588832

**Published:** 2021-03-20

**Authors:** Apa Juntavee, Niwut Juntavee, Praewpan Hirunmoon

**Affiliations:** ^1^Division of Pediatric Dentistry, Department of Preventive Dentistry, Faculty of Dentistry, Khon Kaen University, Khon Kaen 40002, Thailand; ^2^Department of Prosthodontics, Faculty of Dentistry, Khon Kaen University, Khon Kaen 40002, Thailand; ^3^Division of Pediatric Dentistry and Biomaterials Research, Faculty of Dentistry, Khon Kaen University, Khon Kaen 40002, Thailand

## Abstract

**Introduction:**

Nanohydroxyapatite (nano-HA) has been utilized as an alternative agent for dental enamel remineralization. This study compared remineralization potential of nano-HA toothpaste (NHT), functionalized tricalcium phosphate toothpaste (TCPT), and fluoride toothpaste (FT) on carious lesions.

**Materials and Methods:**

Sixty extracted human premolars were prepared for artificial carious lesions with synthetic polymer gel. Samples were divided into four groups according to testing agents: NHT, TCPT, FT, and one group with no treatment (NT). Each group was subjected to pH-cycling with the application of toothpaste in slurry form twice a day (2-min each) for 10 days. Surface microhardness was measured before demineralization, after demineralization, and after pH-cycling. Hardness at different periods, percentage of hardness recovery (% HR), and percentage of remineralization potential (%RP) were determined and statistically analyzed with ANOVA and Tukey comparisons (*α* = 0.05). Polarized light microscopy (PLM) was utilized to assess lesion depth.

**Results:**

Significant remineralization of carious lesions was observed among different toothpastes compared to NT (*p* < 0.05). No significant difference in remineralization potential was found among NHT, TCPT, and FT (*p* > 0.05). No significant difference in % HR and % RP was seen among NHT, TCPT, and FT (*p* > 0.05). PLM indicated a greater decrease in carious depth upon using NHT compared to TCPT and FT, with minimal increase in depth for NT.

**Conclusions:**

NHT has comparable capability to TCPT and FT in hardness recovery. However, decrease in carious depth was evidenced with PLM for NHT more than TCPT and FT. Thus, NHT was suggested as a potential remineralization product for treating initial carious lesions. *Clinical Significance*. The study showed that NHT had the potential to remineralize artificial carious lesion. It was confirmed in potential in the lesion depth reduction and forming a new enamel layer. NHT showed its capability as an alternative for dental caries therapeutic.

## 1. Introduction

One of the most common diseases that has affected the oral health of people around the world is dental caries. It can cause discomfort and pain, leading to limitation in terms of functionality as well as compromised facial aesthetics [1]. It is an energetic alternating cyclic mechanism naturally occurs between demineralization and remineralization of the mineral phase on the tooth surface [2]. The predominance of the demineralization process can lead to tooth cavitation. Carious lesion can be progressed or reversed depending on the balance between pathologic factors, including bacteria, saliva dysfunction, fermentable carbohydrates, and protection mechanism of antibacterial and remineralization agents in sufficient salivation. Initial caries constitutes the preliminary evidence of dental caries, where the enamel surface still remains intact while the subsurface layer is demineralized, thereby leading to cavitation in case no intervention is initiated. In the first phase of enamel demineralization, the aprismatic mineral content is removed, which is subsequently followed by a well-defined surface layer formation that accounts for an early caries lesion [3]. The enamel demineralization process is slow in its progression, which permits the possible reversal of mechanism if the process is detected early and managed adequately. Recently, the conservative approach in dentistry has been applied to the remineralization process as an optimal method to reconstruct the tooth structure. Early identification of caries, together with a conservative treatment of the incipient carious lesion [4], can reduce a number of oral health care-related expenses. Recent research has turned to searching for advanced methods to detect caries at the earliest stage in order to make the use of a noninvasive treatment approach possible.

The demineralization of enamel involves the loss of mineral ions from the hydroxyapatite (HA) crystalline structure at the surface and subsurface of the enamel, leading the acid ions from bacterial plaque to attack the enamel surface [5]. The procedure of repairing the HA crystals using mineral ions is defined as remineralization. It is a natural repairing mechanism for reinstalling to the HA crystalline structure the minerals ions lost. The execution of this process depends upon at the neutral pH of physiologic situations by which the calcium ion (Ca^2+^) and phosphate ions (PO_4_^2−^) from oral fluid are reinserted into the carious lesion, aiding the formation of new fluorapatite that are quite more resistant to acid dissolution [6]. Basically, the demineralization-remineralization phenomenon occurs concurrently at the enamel surface, and a considerable quantity of mineral ions are lost from the HA crystal structure without any destruction of its integrity. When the demineralization process is predominant, it leads to the impairment of the HA lattice integrity and eventually engenders cavitation. However, subsurface demineralization is considered a reversible process. Partial demineralization of the HA crystal structure can be improved and restored if there is sufficient exposure to oral conditions that promote remineralization. The de- and remineralization processes are regulated by the amount of saturation of apatite minerals in oral fluids. Given appropriate oral conditions, it is possible for remineralization to gain predominance, rendering the repairable stage for the lesion. Upon the increase of protective factors, the process can tilt in balance toward remineralization and the initial caries lesion can be checked and reversed [7]. In order to conduct remineralization of the lesion, an increase in the amount of Ca^2+^ or fluoride ion (F^−^)in oral fluids seems necessary to substitute the mineral loss. In view of all this, remineralization has been suggested as the potential approach for caries management nowadays [8, 9].

Fluoride has been recognized as a significant promoter of remineralization, and it has been used to promote fluorapatite formation, which increases the acid resistance capacity of tooth enamel [10]. Fluoride inhibits mineral loss from the enamel surface by inducing surface adsorption on the partially demineralized crystals and attracting Ca^2+^ and PO_4_^2−^ ions from oral fluid as well as forming fluorapatite on enamel surface, thereby increasing enamel resistance to acid attack. Although fluorapatite is more stable and has greater acid resistance than hydroxyapatite, limited remineralization is possible, owing to the amount of Ca^2+^ and PO_4_^2−^ ions available in the oral environment [11, 12]. Fluoride only attempts to minimize the dissolution of apatite rather than promoting mineralization to compensate for the mineral loss in the apatite crystal [13]. It is also found to have significant effectiveness on the smooth surface of the enamel but comparatively less on groove and pits [14]. High initial rates of fluorapatite deposition on the surface layer can inhibit the diffusion of ions into the underlying lesion, thus, leading to the failure to carry out full remineralization [15]. The fluoride intakes during tooth development also increase the risk of dental fluorosis [16]. Hence, it is challenging to search for an alternative and effective nonfluoride agent that can enhance remineralization without the many risks associated with fluoride.

HA is extensively used as bone grafting material, scaffolds for bone tissue engineering, dental implant coatings, soft tissue repair material, desensitizing, remineralization agents, drugs, and gene delivery [17]. The basic foundation of the enamel unit is found to be composed of HA particle, sized 20 to 40 nm. When enamel reaches its maturation, the proteins are almost completely degraded or removed, which leads to the crystallization of apatite; therefore, the enamel cannot be biologically remodeled [18]. It has been suggested that synthetic HA should be used in the remineralization process. The nano-HA formed as a result has been found to possess similar morphology, structure, and crystallinity as a biological apatite. It additionally constitutes a good biocompatible material that is nontoxic and has much higher bioactivity and enhanced mechanical property than larger HA while also having higher resorption [19, 20]. Nano-HA may also act as a reservoir for calcium and phosphate. In acidic conditions, nano-HA is capable of significantly increasing the degree of remineralization by aiding more ions diffusion in the center of the demineralized zone [21]. It has the ability to repair enamel and even prevent the progression of initial lesions owing to the size and the amount of the Ca^2+^ and PO_4_^2−^ ions available. The nanosized particles can directly fill up any small porosity on demineralized surfaces and act as a scaffold for the precipitation that attracts calcium and phosphate from saliva to the enamel surface for the formation of a new apatite layer [22]. It can also adhere to the pores created by demineralization and form a uniform apatite layer. This layer is able to completely cover the prismatic and interprismatic enamel. Nano-HA has also shown a strong affinity toward enamel and appears be a potent biocompatible and bioactive material that has achieved extensive acceptance in medicine and dentistry in recent years [23].

The nano-HA toothpaste can promote remineralization comparable to fluoride toothpaste and, hence, inhibit the progression of demineralization. It was capable of reducing the rate of carious progression and increasing the surface hardness of enamel [24]. An incorporation of nano-HA into sodium fluoride mouth rinse provided a synergistic effect on the remineralization of demineralized enamel lesions [25]. Tricalcium phosphate is a compound material generated with a pulverizing process of fused beta-tricalcium phosphate (*β*-TCP) and sodium lauryl sulfate (SLS) or fumaric acid. This commixture produces a “functionalized” calcium and a “free” phosphate, thereby accelerating the capability of fluoride remineralization. When the functionalized tricalcium phosphate is introduced in the toothpaste alongside fluoride ions, without premature TCP–fluoride interactions, the TCP comes into contact with the moistened tooth surface and the protection layer is broken, resulting in the availability of Ca^2+^, PO_4_^2−^, and F^−^ ions. This phenomenon was designed to increase the efficacy of fluoride remineralization [26]. Previous studies have shown that a functionalized tricalcium phosphate toothpaste (TCPT) has more remineralization effect than a fluoride toothpaste 1,000 ppm [27]. A few studies have been conducted to the remineralization capability of nano-HA toothpaste on artificial carious enamel. This study aimed to compare the remineralization capability of nano-HA toothpaste for artificial carious lesions with tricalcium phosphate containing toothpaste and fluoride toothpaste. The null hypothesis was that the tested toothpastes had no significant capability for remineralization of demineralized enamel. The remineralization potentials of NHT, TCPT, and FT did not have significant differences under the simulated high risk of carious lesion.

## 2. Materials and Methods

This study was endorsed by Khon-Kaen University Ethical Committee in Human Research (Approved No: HE 622235) for the protection of human and animal subjects, and it followed the CRIS guidelines for in vitro studies.

### 2.1. Sample Preparation

**Figure 1:**
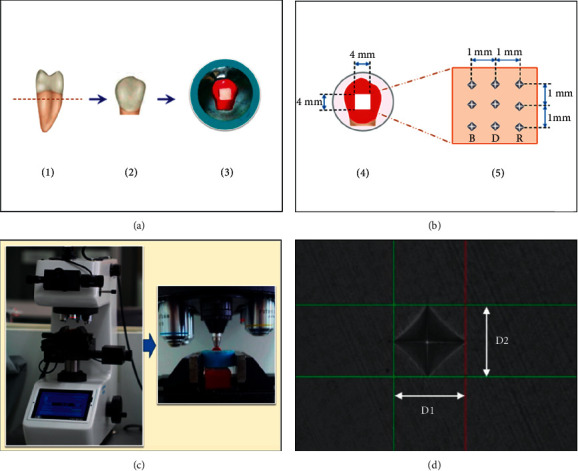
(a) Human second bicuspid was horizontally sectioned at 1 mm below cement enamel junction (1). The crown (2) was invested in the acrylic block (3) to create flat surface area (b) of 4 × 4 mm^2^. The indentation was performed on the flat surface (4) by diamond indenter at 1 mm apart from each other (5) in the microhardness tester (c). The indentation (d) were measured for diagonal length (D1, D2) and calculated for Vicker hardness number.

**Figure 2:**
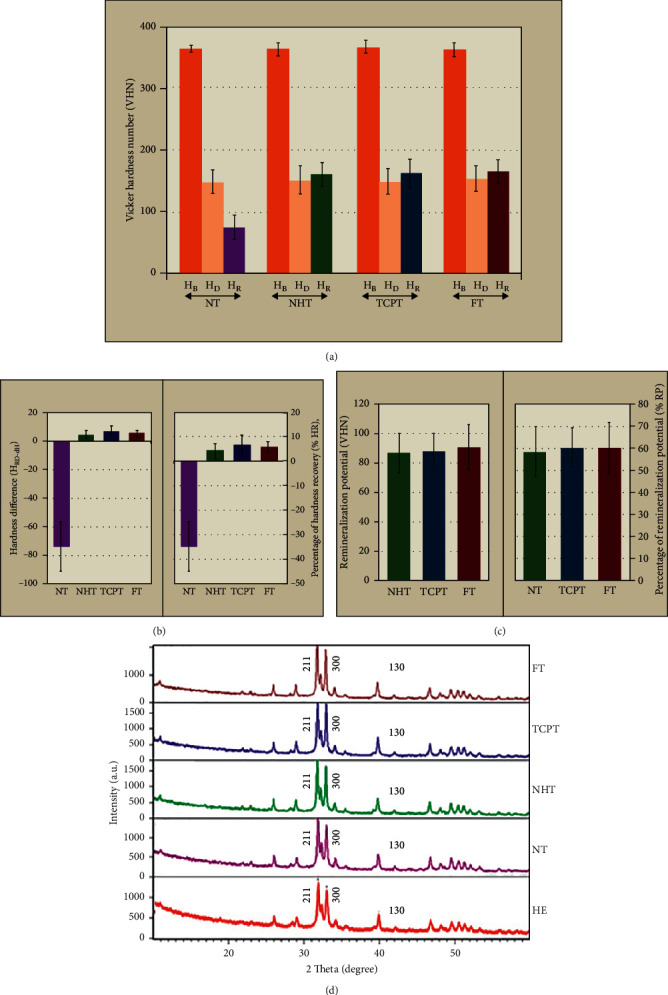
(a) Mean, standard deviation of baseline hardness (*H*_*B*_), and hardness after artificial formation of demineralization (*H*_*D*_). Hardness after application of pH-cycling (*H*_*R*_), (b) hardness difference determined between after application of pH-cycling and after artificial formation of demineralization (*H*_diff_ = *H*_*R*_ − *H*_*D*_), percentage of hardness recovery (% HR), (c) remineralization potential (RP), percentage of remineralization potential (% RP), and (d) XRD patterns of each group treated with nanohydroxapatite toothpaste (NHT), tricalcium phosphate toothpaste (TCPT), and fluoride toothpaste (FT) in comparison to no-treatment group (NT) and healthy enamel (HE).

**Figure 3:**
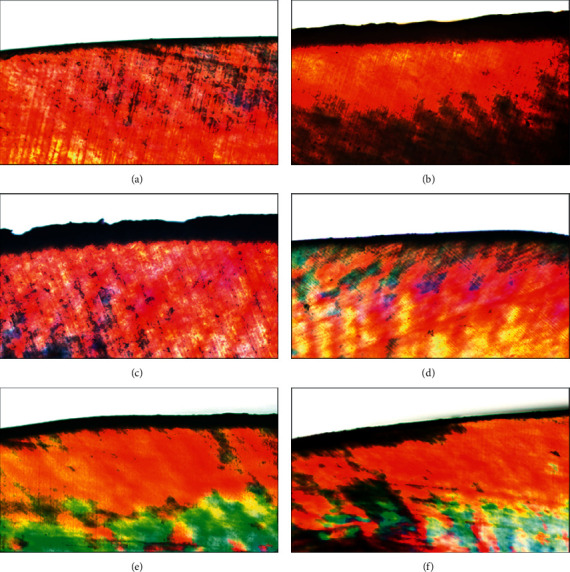
Polarized light micrograph (PLM) at x10 of enamel (a), enamel after artificial formation of demineralization (b), demineralized enamel after application of pH-cycling without remineralization agent (c) and with remineralization agent either nanohydroxyapatite toothpaste (d), or tricalcium phosphate toothpaste (e), or fluoride toothpaste (f).

**Figure 4:**
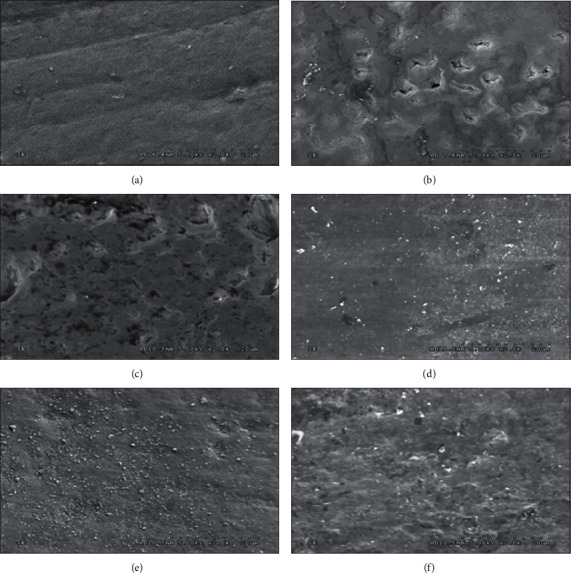
Scanning electron microscopy (SEM) at x2K of enamel (a), enamel after artificial formation of demineralization (b), demineralized enamel after application of pH-cycling without remineralization agent (c) and with remineralization agent either nanohydroxyapatite toothpaste (d), or tricalcium phosphate toothpaste (e), or fluoride toothpaste (f).

Sixty extracted human premolars with orthodontic treatment indication, without dental caries, white spot lesions, fluorosis, cracks, abrasions, fractures, or any developmental anomalies, were selected for the study. Informed consent was obtained from patients and guardians prior to any extraction. The specimens were kept in a 0.1% thymol solution (Chem-supply, Gillman, Australia) until they needed to be used. To separate the crowns from the roots [Figure 1(a)-(1)], the teeth were cut with the precision cutting machine (Mecatome T180, Presi, Eybens, France). The crowns [Figure 1(a)-(2)] were embedded in an epoxy resin block with the enamel above being exposed to the epoxy resin. The surface of the enamel was covered with an acid-resistant varnish (Revlon, New York, NY, USA) except for a surface area of 4 × 4 mm [Figure 1(a)-(3)]. The unpainted area of enamel was ground flat with a silicon carbide abrasive (Buehler, Tokyo, Japan) # 1,000, 2000, and 4000 grit, respectively, in a polishing machine (Ecomet, Buehler, Tokyo, Japan) [Figure 1(b)-(4)]. The specimens were then cleaned and kept in 37°C deionized water for 24 hours.

### 2.2. The Induction of Artificial Carious Lesion

The gel for inducing the artificial carious lesion was prepared for the demineralization process to comprise 20 grams/L of Carbopol-907 (BF-Goodrich, Cleveland, OH, USA), 500 mg/L of HA, 0.1% of lactic acid, and adjusted-pH to 5.0 by sodium-hydroxide [28]. The specimens were submerged in an individual sterile container containing demineralization gel and kept in a humidified environment for 12 hours, following which, they were cleaned with deionized water in order to produce a consistent subsurface artificial carious lesion on the enamel.

### 2.3. The Application of Acid Challenge Model of pH-Cycling with Tested Toothpastes

The specimens were randomly grouped (*n* = 15) prior to manipulation in the acid challenge model of pH-cycling and remineralization procedure with different remineralization materials, shown in Table 1, as follows:

  Group NHT: treated with the nano-HA toothpaste (Apagard®, Sangi, Tokyo, Japan)
  Group TCPT: treated with the functionalized TCPT (Clinpro™, 3M, St Paul, MN, USA)
  Group FT: treated with fluoride toothpaste (Colgate®, Colgate-Palmolive, Cholburi, Thailand)
  Group NT: left untreated in the deionized water and served as a control group

The pH-cycling model consisted of demineralizing- and remineralizing-solution as well as artificial saliva [29]. The demineralizing agent contained 2.2 mM CaCl_2_, 2.2 mM KH_2_PO_4_, and 0.05 M CH_3_COOH, and its pH is adjusted to 4.4 with 1 M KOH. The remineralizing agent contained 1.5 mM CaCl_2_, 0.9 mM NaH_2_PO_4_, and 0.15 M KCl, and the pH is adjusted to 7.0. The artificial saliva contained 0.650 g/L KCl, 0.058 g/L MgCl_2_, 0.165 g/L CaCl_2_, 0.804 g/L K_2_HPO_4_, 0.365 g/L KH_2_PO_4_, 2 g/L C_6_H_5_COONa, 7.80 g/L C_8_H_15_NaO_8_, and the pH is adjusted to 7.0 [30]. The solutions were freshly prepared for each cycle. All specimens were stored individually in 10 ml of each solution in scintillation vials and placed in the pH-cycling and remineralization process system in a shaking water bath (Wise Bath, Seoul, Korea) at 37°C for 10 days. The pH-cycle comprised three hours of demineralization, with two hours of remineralization twice a day and submerging in artificial saliva for 14 hours. Each toothpaste was prepared in a slurry form by mixing 15 g of toothpaste with 45 ml of deionized water [31]. The experimental groups were immersed in 5 ml solution of slurry toothpaste for two minutes, before the first demineralization and after the second remineralization, and rinsed with deionized water after each application.

### 2.4. The Determination of Surface Hardness

The microhardness was evaluated prior to the application of demineralized solution (*H*_*B*_), after inclusion in the demineralization process (*H*_*D*_) and after treatment in the pH-cycling and remineralization process (*H*_*R*_). Each time of measurement was evaluated randomly at three points, and measurements were taken at 1-mm indentation apart from one another [Figure 1(b)-(5)]. Vickers indenter of 100 g load for 15 seconds of lodging time with a digitalized hardness testing machine (Future-tech, Tokyo, Japan) was used, as shown in Figure 1(c). The diagonal lengths of the indenter's imprint (D1, D2) were measured [Figure 1(d)] and calculated for Vickers hardness number (VHN). Hardness differences (*H*_diff_) were further calculated for the percentage of surface hardness recovery (% HR) and the percentage of remineralization potential (% RP) were performed according to the following equations:

(1) $$\begin{array}{c} \%\text{ HR}=\frac{H_{R}-H_{D}}{H_{B}-H_{D}}\times 100, \end{array}$$

(2) $$\begin{array}{c} \%\text{ RP}=\frac{H_{R}-H_{R^{*}}}{H_{D}}\times 100, \end{array}$$

where *H*_*R*^*∗*^_ refers to hardness upon the application of pH-cycling and remineralization to the no-treatment group.

### 2.5. Microscopic Evaluation

The specimen from each group was longitudinally sectioned for 200 *µ*m thickness, cleaned with deionized water, and further evaluated under a polarizing light microscopy (PLM, Eclipse80i, Nikon, Kanagawa, Japan) at 10x magnification. The PLM of the normal intact enamel surface and demineralized enamel surface were then performed at 10x magnification and used as references in comparisons with experimental groups.

The specimen from each group was gold-coated in a sputtering machine (Emitech-K500X, Quorum, Asford, UK) and analyzed for surface alteration in the scanning electron microscope (SEM, S-3000N, Hitachi, Tokyo, Japan) in comparison with the SEM photomicrograph of the intact enamel and demineralized enamel at X2.0K.

The samples from each group, healthy enamel (HE), and demineralized enamel (DE) were randomly selected and pulverized into finely ground particles. They were then further examined for the crystalline structure with the help of an X-ray diffractometer (PANalytical BV, Almelo, Netherland). The fine particles were scanned with copper k-alpha (Cu K*α*) radiations at 40 kV, 30 mA, between the 2*θ* degree of 10–60°. The crystal structures were determined by comparing with the reference data of powder diffraction and justified for the intensity of the peaks with software (X'pert plus, PANalytical BV, Almelo, Netherland) at step size of 0.02° for each 2 seconds. Then, the crystal size was determined using Scherrer's formula, as follows:

(3) $$\begin{array}{c} D=\frac{K\lambda }{B\mathrm{Cos}\Theta }, \end{array}$$

where *D* is the average crystal size, *K* is the Scherrer constant, *λ* indicates the wavelength, *B* represents the width at half maximum, and Ɵ is the peak's position.

### 2.6. Statistical Analysis

The data were determined with the help of SPSS-v. 19 (IBM-SPSS, Armonk, New York, USA). An analysis of variance (ANOVA) was performed to determine the significant difference of Vickers microhardness at different stages of determination, including *H*_*B*_*, H*_*D*_*, H*_*R*_, *H*_diff_, % HR, RP, and % RP of the enamel. Post hoc Tukey HSD multiple comparisons were made to determine a statistical significance among the groups at *α* = 0.05.

## 3. Results

The mean, standard deviation (sd), and 95% confidential interval (CI) of the *H*_*B*_, *H*_*D*_, *H*_*R*_, *H*_diff_, % HR, RP, and % RP for each group are shown in Table 2 and Figures 2(a)–2(c). ANOVA and post hoc Tukey multiple comparisons revealed no significantly different *H*_*B*_ among the tested groups (*p* > 0.05), as indicated in Table 3 (A) and Table 4 (A). The mean *H*_*D*_ was decreased for each group compared to *H*_*B*,_ as shown in Figure 2(a). However, no significantly different *H*_*D*_ was indicated among the tested groups (*p* > 0.05), as displayed in Table 3 (B) and Table 4 (B). Upon the application of the pH-cycling and remineralization agent, the mean *H*_*R*_ was significantly increased for each group compared to *H*_*D*_ (randomly), except for the NT group (*p* > 0.05), as shown in Figure 2(a). The mean *H*_*R*_ was significantly different as a result of remineralizing products tested, as shown in Figure 2(a) and Table 3 (C). Post hoc Tukey multiple comparisons indicated significant differences in the mean *H*_*R*_ among the tested group (*p* < 0.05), except for FT-NHT, FT-TCPT, and NHT-TCPT, as shown in Table 4 (C). ANOVA indicated a significant difference in the mean H_RD-diff_ among the tested groups (*p* < 0.05), except for FT-NHT, FT-TCPT, and NHT-TCPT, as shown in Figure 2(b), Table 3 (D), and Table 4 (D). Significant differences in the mean % HR among the tested groups (*p* < 0.05) were noted, except for FT-NHT, FT-TCPT, and NHT-TCPT, as shown in Figure 2(b), Table 3 (E), and Table 4 (E). The highest percentage of hardness recovery was revealed for the group that underwent treatment with TCPT, followed by the FT group and the NHT group. ANOVA indicated no significant difference in the mean RP amid the groups (*p* > 0.05), as indicated in Figure 2(c), Table 3 (F), and Table 4 (F). No significant differences in the mean % RP among groups (*p* > 0.05) were indicated, as revealed in Figure 2(c), Table 3 (G), and Table 4 (G). The remineralization potentials of TCPT, FT, and NHT were comparable. The application of TCPT, FT, and NHT indicated significant capability for remineralization of the demineralized enamel (*p* < 0.05), but their capability of remineralization was comparable.

The XRD analysis indicated crystalline structure at the 2*θ* degree of 26°, 29°, 31°, 32°, 33°, 34°, 40°, 47°, 49°, 50°, and 51°, coinciding with the plane (002), (210), (211), (112), (300), (202), (130), (222), (213), (321), and (140), respectively, for every group, as shown in Figure 2(d). The observed peaks for all groups were consistent with the standard crystal structure of hydroxyapatite and fluorapatite. The peak exhibited a well-defined sharp peak, with different peak intensity observed among the groups. The highest peak intensity was indicated at the 2*θ* degree of 31°, 33°, and 40°, which coincided with the planes (211), (300), and (130), as shown in Figure 2(d). The position of the observed peaks is almost similar for all groups treated with toothpastes, which indicated the capability of generating more complex degree of crystallinity compared to the NT group. This showed that the samples have similar planes of atoms, sizes, and shapes of unit cell. The three toothpastes exhibited a well-defined sharp peak. This indicated that the three toothpastes have a higher degree of crystallinity. The NT group was observed with broad and short peaks compared to the HE and DE, indicating the formation of artificial carious lesions, as shown in Figure 2(d). This shows that the NT group had amorphous and poorly crystallized apatite. The average crystalline size for all the groups was within the range of 34.80 to 46.77 nm. The largest crystallite size was observed for the group of TCPT (46.77 nm), followed by FT (39.49 nm), NHT (37.07 nm), and NT (34.80 nm) groups.

The presence of a carious lesion and the progression of the remineralization process for each tested group are presented with the PLM in Figure 3 in comparison to the PLM for intact enamel without evidence of the carious lesion, as shown in Figure 3(a). An obvious dark area and increased lesion depth were observed on the PLM of the artificial carious specimen (Figure 3(b)). After the application of pH-cycling and remineralization process, the reduction of lesion depth in all groups treated with tested toothpastes is shown in Figures 3(d)–3(f), but increasing of lesion depth for the NT group is shown in Figure 3(c) compared to the original lesion depth upon artificial demineralization (Figure 3(b)). This indicated that all the experimental toothpastes were capable of remineralization on the artificial carious lesion. It was observed that the carious lesion for NHT group has greater lesion depth reduction than the TCPT and FT groups. The NT group showed increasing lesion depth, indicating that no remineralization was presented after the pH-cycling process.

The SEM photomicrograph at x2K magnification for each treatment group was observed in comparison with the SEM photomicrograph of intact enamel as shown in Figure 4(a). The artificial induced carious sample indicated an irregular pattern of slits and surface destruction with high evidence of porosities as observed in Figure 4(b) compared to the SEM micrograph of smooth and intact normal enamel surface as observed in Figure 4(a). The SEM photomicrograph for the NT group showed more porosity and roughness surface, which indicated no remineralization presented after the pH-cycling process (Figure 3(c)). The SEM photomicrograph for the NHT specimen showed smooth and homogeneous surface, indicating the formation of a new apatite layer and remineralization of the carious lesion (Figure 4(d)). The SEM photomicrograph for the TCPT specimen exhibited a slight roughness and micropore surface in some areas, which indicated that there were some formations of fluorapatite in the demineralized surface but not complete remineralization (Figure 4(e)). The SEM photomicrograph for the FT group indicated thin layer of mineralization with an incomplete filling of the voids and porosities generated from the previously induced carious lesion, as shown in Figure 4(f), which indicates incomplete remineralization capability of FT over the entire surface of the carious lesion induced on the enamel surface. The SEM photomicrographs for the groups of NHT revealed a smooth and homogeneous enamel surface, similar to that from the TCPT and FT groups. The SEM photomicrographs indicated a formation of a new apatite layer and remineralization presented on the induced carious enamel treated with NHT, TCPT, and FT. In contrast, deeper porosities and a more prominent irregular pattern of destruction on the SEM photomicrograph of the induced carious lesion of the enamel surface for the NT group were observed, as shown in Figure 4(c).

## 4. Discussion

The remineralization of initial carious lesions can be considered as a preventive means and a therapeutic approach in restorative dentistry in the following decade. This study evaluated the abilities of NHT, TCPT, and FT for the remineralization of the carious enamel. The study indicated that NHT, TCPT, and FT had significant capability for remineralization in recovering the demineralized enamel compared with the nontreated demineralized surface (NT group). This means that the experimental toothpastes were capable of remineralization in the artificial demineralized enamel. However, there was no significance difference in the remineralization efficacy among the three groups of toothpastes, and the null hypothesis was accepted for the remineralization potential of the toothpastes. The PLM illustrated decrease in carious lesion depth upon treating the demineralized surface with three experimental toothpastes in comparison to the NT group with slightly increased in lesion depth due to acid challenge of the pH-cycling model. Nevertheless, the remineralization capability of toothpastes was also supported by the XRD, exhibiting higher intensity of peak crystalline structure on the demineralized surface in comparison to the NT group. In addition, the NHT group indicated superior capability in remineralization of the demineralized surface to both TCPT and FT compared to NT group, as evidenced by SEM.

Nanohydroxyapatite containing toothpastes have been newly introduced in the market. At present, toothpastes containing 10% nano-HA as optimal concentration are used for remineralization of the initial carious lesion [22]. In a previous study, it was found that the percentage of surface hardness recovery of 10% NHAT and 1,000 ppm FT was not a significant difference, and this was also supported by other studies [23, 24]. In contrast, higher remineralization potential for NHT, compared to FT, was found in our study, which contradicts other studies [16]. This may be attributed to the difference in experimental design, treatment regimens, sample preparation, equipment used, and data interpretation.

For SEM analysis, NHT showed smooth, homogeneous surface with no porosity, while FT showed roughness of surface and slight porosity. This might be due to the characteristics of nanohydroxyapatite, with similarity in enamel structure that can create the induction of initial caries remineralization upon generating a homogeneous apatite complex structure on the demineralized enamel once the toothpaste is applied [13]. This complex structure comprises synthetic hydroxyapatite bonding directly with natural crystalline enamel. Thus, the hardness of the remineralized enamel is comparable with natural enamel [10]. In contrast, the remineralization of enamel after FT application requires calcium and phosphate ions from the solution to form fluorapatite on the demineralized enamel, and with the limitation of these inorganic compounds in saliva, the remineralization process cannot be completed [8]. Thus, the SEM images confirm that NHAT showed more remineralizing potential than FT. In addition, PLM analysis demonstrated that NHAT provided greater reduction of lesion depth in comparison with FT due to nano-crystals, which can be aggregated in the skeleton of carious lesions, and eventually reduced the lesion depth [21]. The study was consistent with the study by Manchery et al. (2019), which compared the remineralization efficacy of 10% NHAT, 5% calcium sodium phosphosilicate, and 1,450-ppm FT under PLM investigation and reported that NHAT has more capability to reduce lesion depth than FT [22].

This study indicated that the percentage of surface hardness recovery of TCPT was comparable to that of FT. This finding was consistent with Vanichvatana and Auychai, which reported similar remineralizing efficacies for TCPT and 1,000 ppm FT [32]. However, SEM and PLM investigations revealed that TCPT had more remineralizing potential than FT, which was also consistent with Buckshey et al. [27]. The NHT group showed better remineralization potential in SEM and PLM than the FT and the control group. The smaller-sized particles and higher activity of the nano-HA probably helps the compound penetrate through the surface of the enamel and continuously fill the porosities of the artificial carious area better than fluoride [21]. In addition, the results of this study indicated that NHT is comparable to FT in terms of increasing surface microhardness for the carious enamel. The other critical factor that might enhance the remineralization potential of the NHT group is an acidic environment of the pH-cycling, since acidic condition increase the solubility of the nano-HA, thereby increasing the deposition of nano-HA on the surface of the enamel [21]. In this study, the pH-cycling was designed to mimic the acid challenge condition of the oral cavity, thus potentiating the results of superior remineralization effects for NHT over TCPT and FT, as demonstrated in the SEM and PLM. All experimental groups, except for the control group, exhibited the reduction of lesion depth, as described by the PLM; nevertheless, complete remineralization was not achieved.

In the FT group, it was postulated that the hydroxyl group was substituted by fluoride ions and the superficial surface layer became very exothermic, while the deeper layer progressively generated less exothermic energy, thereby leading the surface hydroxyl group to be easily replaced by fluoride ions [11]. As such, the outer surface layer became highly mineralized with so-called hypermineralization, and this decreased the capability of the mineral to penetrate into deeper layers, as shown in the PLM photomicrograph in the FT group of this study. SEM photomicrographs demonstrated that NHT was able to form a homogeneous apatite surface layer, and this coincides with the findings of previous studies [3]. The enamel which was covered by a homogeneous layer of chemical binding of synthetic HA to natural enamel may facilitate the formation of a new apatite layer [13]. In addition, strong affinity between nano-HA and enamel was possible due to the crystallinity of calcium and phosphate that demonstrated a crucial role in the process of enamel mineralization.

The observed peaks for all groups in XRD analysis showed a similar crystal structure as the one found in natural HE. In the FT and TCPT group, it was not possible to distinguish between the hydroxyapatite and fluorapatite peak, as the positions were overlapped [4, 14]. Sharper peaks observed in the FT group indicated high crystallinity that is possible due to the conversion of hydroxyapatite into fluorapatite [13]. Narrow peaks of NHT indicated a smaller crystalline size of toothpaste formulation, which was confirmed using Scherrer's equation. The broadening peak that was observed in the NT group possibly resulted from the partial dissolution of the individual crystals, leading to the enlargement of the intercrystalline spaces, thus decreasing their crystalline size [12].

The microhardness determination is related to the strength of enamel that resulted from the combination of mineral densities bonding within enamel structure. All the experimental toothpastes showed no significant difference in terms of microhardness. The qualitative analysis of the PLM showed that NHAT and TCPT provided greater reduction of lesion depth and demonstrated more particle deposition in the demineralized enamel surface in SEM rather than FT. The pH-cycling was designed to mimic the acidic-challenged condition and simulate dynamic mineral saturation associated with the natural caries process [28]. This pH-cycling model is effectively designed to evaluate lesion progression based on crystal changes and the microhardness of the enamel [29]. Artificial carious lesions in this study were fabricated with the help of synthetic polymer gel. This gel comprised polyacrylic acid to preserve the surface layer and produce sub-surface caries formation, similar to the natural initial carious lesion [28]. The limitations of the study are those associated with an experimental study that has limited capability to simulate plaque, saliva pellicle, and bacterial biofilms present in oral cavity. Moreover, each of the sample may word in a different manner based on the donor's age and environmental exposure to diet. These limitations lead to variation in responses under acidic-challenged conditions. Furthermore, the experimental periods of demineralization and remineralization were less than the expected period in vivo conditions [29]. Toothpaste containing nano-HA or functional tricalcium phosphate is considered as a novel biocompatible product [4, 20] that can provide more cost-effectiveness therapeutics to dental caries in un-cooperative children and/or medically compromised patients who may otherwise have trouble with oral hygiene control. Further scientifically experimental studies are recommended for in situ clinical trials. Nevertheless, this study was definitely crucial as a primary evaluation of new toothpaste products, in order to provide valuable data for clinicians and researchers to pursue effective clinical trials in the future.

## 5. Conclusion

This in vitro study designated the potential remineralization of nano-HA toothpaste to initial carious lesion comparable to TCPT and FT. From the SEM, PLM, and XRD investigation; NHT and TCPT exhibited more remineralization potential than FT upon the remineralization of carious enamel. NHT and TCPT could be considered as preventive and therapeutical agents for initial carious lesion. Further clinical trials are needed to confirm the remineralization potential of these toothpastes.

## 6. Statement of Significance

Remineralization therapy is a contemporary approach for carious lesions detecting at the earliest stage of the disease to render a noninvasive treatment approach that is crucial for dental profession in shifting their therapeutic approach to a new paradigm. The present study showed that nano-HA toothpaste had the potential to remineralize the artificial carious lesion. It was confirmed for potential lesion depth reduction and forming a new enamel layer. The nano-HA toothpaste showed its capability as an alternative for dental caries therapeutic especially in un-cooperative young children and in medically compromised patients.

## Figures and Tables

**Table 1:** Materials, company, compositions, and batch number of toothpastes used in this study.

Materials	Company	Composition	Batch number
Nano-HA toothpaste (NHT)	Apagard® Royal, Sangi Co., Tokyo, Japan	10% nanohydroxyapatite, glycerin, PEG-400, ß-glycyrrhetinic acid, sodium lauryl sulfate, sodium carboxymethylcellulose, sodium saccharin, hydrolysed conchiolin solution, trimagnesium phosphatemedical	ZA02
Tricalcium phosphate toothpaste (TCPT)	Clinpro™ Tooth Crème, 3M ESPE, St Paul, MN, USA	Tricalcium phosphate, sodium lauryl sulfate, sorbitol, hydrated silica, glycerin, poloxamer 407, aroma, PEG-12, titanium dioxide, cellulose gum, sodium saccharin, 0.21% sodium fluoride	11001
Fluoride toothpaste (FT)	Colgate® regular flavor, Colgate-Palmolive, Chonburi, Thailand	0.22% sodium monofluoride, dicalcium phosphate dihydrate, water, sorbitol, sodium lauryl sulfate, flavor, tetrasodium pyrophosphate, cellulose gum, sodium saccharin	TH112B

**Table 2:** Mean, standard deviation (sd), 95% confidential interval (CI) of baseline hardness (*H*_*B*_), hardness after artificial formation of demineralization (*H*_*D*_), hardness after application of pH-cycling and remineralization process (*H*_*R*_), hardness difference determined between after application of pH-cycling and remineralization process and after artificial formation of demineralization (*H*_diff_ = *H*_*R*_ − *H*_*D*_), percentage of hardness recovery (% HR), remineralization potential (RP), percentage of remineralization potential (% RP), and average crystal size (CS) for no treatment (NT), nanohydroxyapatite toothpaste (NHT), tricalcium phosphate toothpaste (TCPT), and fluoride toothpaste (FT) group.

Group	*H*_*B*_	*H*_*D*_	*H*_*R*_	*H*_diff_ = *H*_*R*_ − *H*_*D*_	% HR	RP	% RP	CS (nm)
	Mean ± sd 95% CI (LL-UL)	Mean ± sd 95% CI (LL-UL)	Mean ± sd 95% CI (LL-UL)	Mean ± sd 95% CI (LL-UL)	Mean ± sd 95% CI (LL-UL)	Mean ± sd 95% CI (LL-UL)	Mean ± sd 95% CI (LL-UL)	
NT	362.06 ± 10.11^a^ (356.46–367.66)	147.07 ± 18.93^b^ (136.59–157.55)	73.38 ± 19.50^c^ (62.58–84.18)	−73.69 ± 17.13^p^(−83.18)–(−64.20)	−34.78 ± 9.96^n^ (−40.30)–(−29.27)	—	—	34.80
NHT	361.65 ± 9.89^a^ (356.18–367.13)	150.77 ± 22.49^b^ (138.31–163.23)	160.29 ± 18.60^d^ (149.99–170.59)	9.52 ± 6.54^h^ (5.90–13.14)	4.39 ± 2.83^m^ (2.83–5.96)	86.91 ± 13.65^n^ (79.36–94.47)	58.62 ± 11.20^p^ (52.42–64.82)	37.07
TCPT	361.54 ± 9.56^a^ (356.26–366.83)	147.55 ± 21.10^b^ (135.87–159.24)	161.78 ± 21.70^d^ (149.77–173.80)	14.23 ± 8.09^h^ (9.75–18.71)	6.69 ± 3.94^m^ (4.51–8.87)	88.41 ± 12.47^n^ (81.50–95.31)	60.61 ± 8.88^p^ (55.70–65.53)	46.77
FT	360.89 ± 11.01^a^ (354.80–366.99)	152.22 ± 20.31^b^ (141.00–163.43)	164.26 ± 19.44^d^ (153.49–175.02)	12.04 ± 4.74^h^ (9.41–14.67)	5.74 ± 2.09^m^ (4.58–6.90)	90.88 ± 15.49^n^ (82.30–99.46)	60.49 ± 11.66^p^ (54.02–66.95)	39.49

NB: different superscript letters in the same column represented significant different between treatment group (*p* < 0.001). LL: lower limit. UP: upper limit.

**Table 3:** An analysis of variance (ANOVA) of baseline hardness (*H*_*B*_), hardness after artificial formation of demineralization (*H*_*D*_), hardness after application of pH-cycling and remineralization process (*H*_*R*_), hardness difference determined between after application of pH-cycling and remineralization process and after artificial formation demineralization (*H*_diff_ = *H*_*R*_ −*H*_*D*_), and percentage of hardness recovery (% HR), remineralization potential (RP), and percentage of remineralization potential (% RP).

Source	SS	df	MS	F	*P*
*A. One-way ANOVA of baseline hardness (H*_*B*_)					
Between group	10.566	3	3.522	0.034	0.991
Within group	5772.199	56	103.075		
Total	5782.765	59			
*B. One-way ANOVA of after demineralization (H*_*D*_)					
Between group	429.841	279.961	3	93.320	0.217
Within group	46035.366	24067.514	56	429.777	
Total	46465.207	24347.475	59		
*C. One-way ANOVA of after application of pH-cycling and remineralization process (H*_*R*_)					
Between group	88696.582	3	29565.527	75.091	0.001
Within group	22048.751	56	393.728		
Total	110745.333	59			
*D. One-way ANOVA of hardness difference between after application of pH-cycling and after demineralization (H*_*diff*_)					
Between group	82643.143	3	27547.714	259.627	0.001
Within group	5941.878	56	106.105		
Total	88585.020	59			
*E. One-way ANOVA of percentage of hardness recovery (% HR)*					
Between group	18392.686	3	6130.895	192.848	0.001
Within group	1780.314	56	31.791		
Total	20173.000	59			
*F. One-way ANOVA of remineralization potential (RP)*					
Between group	120.300	2	60.150	0.310	0.735
Within group	8146.985	42	193.976		
Total	8267.285	44			
*G. One-way ANOVA of percentage of remineralization potential (%RP)*					
Between group	302.066	2	18.748	0.165	0.848
Within group	88397.405	42	113.492		
Total	88699.471	44			

SS: sum of squares, df: degree of freedom, MS: mean square, F: *F* ratio, *p*: *p* value.

**Table 4:** Post hoc Tukey's multiple comparisons of baseline hardness (*H*_*B*_), hardness after artificial formation of demineralization (*H*_*D*_), hardness after application of pH-cycling and remineralization process (*H*_R_), hardness difference determined between after application of pH-cycling and remineralization process and after artificial formation demineralization (*H*_diff_ = *H*_*R*_ − *H*_*D*_), percentage of hardness recovery (% HR), remineralization potential (RP), percentage of remineralization potential (% RP) among no treatment (NT), nanohydroxyapatite toothpaste (NHT), tricalcium phosphate toothpaste (TCPT), and fluoride toothpaste (FT) group.

Group	NT	NHT	TCPT	FT
*A. Tukey HSD multiple comparison of baseline hardness (H*_*B*_)				
NT	1	1.000	0.999	0.989
NHT		1	1.000	0.997
TCPT			1	0.998
FT				1
*B. Tukey HSD multiple comparison after artificial formation of demineralization (H*_*D*_)				
NT	1	0.961	1.000	0.904
NHT		1	0.974	0.997
TCPT			1	0.926
FT				1
*C. Tukey HSD multiple comparison of after application of pH-cycling and remineralization process (H*_*R*_)				
NT	1	0.001	0.001	0.001
NHT		1	0.997	0.947
TCPT			1	0.986
FT				1
*D. Tukey HSD multiple comparison of hardness difference (H*_*diff*_ *) between H*_*R*_ *and H*_*D*_				
NT	1	1	0.001	0.001
NHT		1	0.598	0.909
TCPT			1	0.937
FT				1
*E. Tukey HSD multiple comparison of percentage of hardness recovery (% HR)*				
NT	1	0.001	0.001	0.001
NHT		1	0.387	0.624
TCPT			1	0.961
FT				1
*F. Tukey HSD multiple comparison of remineralization potential (RP)*				
NHT		1	0.954	0.717
TCPT			1	0.878
FT				1
*G. Tukey HSD multiple comparison of percentage of remineralization potential (% RP)*				
NHT		1	0.865	0.881
TCPT			1	0.999
FT				1

## Data Availability

The data used to support the findings of this study are included within the article.
